# Post-Traumatic Stress Disorder Among Undocumented Immigrants. Evidence for the Premier-Pas Survey

**DOI:** 10.3389/ijph.2026.1608844

**Published:** 2026-04-15

**Authors:** Constance Prieur, Vincent Lhote, Antoine Marsaudon, Stéphanie Guillaume, Florence Jusot, Jérôme Wittwer, Paul Dourgnon

**Affiliations:** 1 Centre Hospitalier Universitaire Vaudois, Lausanne, Switzerland; 2 Universite Paris-Pantheon-Assas, Paris, France; 3 LIRAES, F-75006, Université Paris Cité, Paris, France; 4 IRDES, Paris, France; 5 Universite Paris Dauphine, Paris, France; 6 Universite de Bordeaux, Talence, France; 7 Bordeaux Population Health, Bordeaux, France

**Keywords:** France, mental health, migration, posttraumatic stress disorder, undocumented immigrants

## Abstract

**Objectives:**

Undocumented immigrants are a highly vulnerable population, frequently exposed to violence and trauma in their country of origin, along the migration journey, and in the host country. This study investigates which factors experienced before, during, and after migration influence the development of post-traumatic stress disorder (PTSD). It also investigates whether PTSD relates to high-risk health behaviors in France.

**Methods:**

We relied on a survey representative of undocumented immigrants attending facilities providing assistance to vulnerable populations in Paris and Bordeaux (France). Analyses relied on several multivariate probit models (N = 1,060).

**Results:**

Overall, 54.1% of respondents experienced at least one traumatic event, and 17.2% currently suffer from PTSD. Factors associated with an increase probability to develop PTSD are: coming to France for safety reasons (before migration), entering France without appropriate documentation (during migration), food insecurity and poor housing conditions (after migration). PTSD is also associated with an increase probability to engage in high-risk alcohol consumption.

**Conclusion:**

Although pre-migration factors cannot be addressed by destination-country policies, our findings suggest that interventions targeting deprivation may help reducing PTSD and substance use among undocumented immigrants.

## Introduction

In 2024, 918, 925 individuals were found to be present in the European Union (EU) without appropriate documentation. Undocumented immigrants were estimated to account for approximately 0.2% of the total European population [1]. According to the European Commission, an undocumented immigrant refers to a person living in “a new place of residence or transit that takes place outside the regulatory norms of the sending, transit and receiving countries” [2]. Some immigrants entered documented but become undocumented by overstaying their visa. Others entered and stayed undocumented in the destination country.

Undocumented immigrants face legal, social, and economic vulnerabilities making the investigation of their mental health particularly important. They are often exposed to violence throughout their migration journey [3], placing them at high risk of developing post-traumatic stress disorder (PTSD). PTSD is a mental and behavioral disorder that can occur after exposure to a traumatic event. It is characterized by re-experiencing, avoidance, hyperarousal, and emotional numbing, with symptoms persisting for weeks or longer, and significantly impairing social functioning [4, 5].

Exposure to trauma can occur at all stages of the migration (that is, before, during, and after) and may be worsened by precarious living conditions such as, inadequate housing, food insecurity, language barriers, and social isolation in the host country [3, 6]. These conditions can exacerbate trauma, deplete physical and psychological resilience, and increase the likelihood of developing chronic PTSD [7, 8, 9].

A large body of the literature showed that refugees experienced high rates of PTSD, depression, and other affective disorders [10–12]. Existing studies investigated the mental health of undocumented immigrants have relied either on small samples, which limits the statistical power of the analysis [13–17] or on larger but selected samples of specific country of origin [18] or drawn from healthcare facilities [19, 20]. Supplementary Table A1 provides a summary of the sampling characteristics of the above-mentioned studies.

This study investigates which factors experienced before, during, and after migration that influence the development of PTSD. It also investigates whether PTSD relates to high-risk health behaviors in France. To do so, we relied on a representative sample of undocumented immigrants attending facilities providing assistance to vulnerable populations in Paris and Bordeaux (France). We believe this dataset allowed us to contribute to the existing literature for the following reasons. First, by scaling-up the number of observations, which allow to control for important confounding factors and to improve the statistical power of the analysis. Second, other datasets surveyed undocumented immigrants in healthcare facilities, thereby introducing a selection bias toward individuals in poorer health. Third, individuals attending assistance facilities represent a particularly relevant population, as they can be more readily identified and reached through targeted public interventions.

## Methods

The empirical analysis relies on data from the “Premiers pas” (first steps) survey. It was designed and conducted by IRDES (Institute for Research and Information in Health Economics, a French research center specialized in health economics), and funded by the French Research National Agency. The survey was launched from February to April 2019 in the greater Bordeaux area and in Paris. The survey protocol followed a two-stage procedure. In the first stage, facilities providing assistance to vulnerable populations were selected—these facilities were attended by immigrants regardless of legal status and by low-income French individuals. These facilities offered various assistance services: administrative supports, food and cloth distributions, hygiene and healthcare services, as well as educational and cultural activities. Among these facilities, 113 mentioned that at least 20 undocumented immigrants came in a typical week, and 63 of them agreed to administer the questionnaire. By focusing on all these services, we extend the existing literature, which has primarily focused on healthcare structures [21, 22]. However, recruiting in facilities providing assistance generate an additional selection bias, as these services may disproportionally attract individuals with higher levels of social and health-related needs.

In the second stage, interviewers (who were chosen to be first- or second-generation immigrants in order to reduce the cultural distance with the respondents. They spoke at least French and English) collected questionnaires in these facilities. They also completed, in a separated document, the characteristics of the facility (i.e., detailing its organization, and any other information that might affect data collection). In each facility, respondents were randomly selected. 49% of these respondents were not within the scope of the survey (because they were French citizens, documented immigrants, or refugees), 8% did not participate owing to language barriers, 42% refused to participate, and 50% agreed.

Questionnaires were displayed in 14 languages. Of those who participated, 75% responded in French, followed by 8% in Arabic, 7% in English, 4% in Spanish, 2% in Russian, and 2% in Portuguese.

To achieve representativeness of the population attending these facilities, survey weights were constructed for each category of facilities. Each facility reported the average number of undocumented immigrants received during a representative week. On the day of the data collection, interviewers also recorded the actual number of undocumented immigrants present at the facility. Weights were then adjusted to correct for discrepancies between the reported weekly average and the actual attendance observed on the interview day. If the number of undocumented immigrants present during the data collection was lower (higher, respectively) than the reported weekly average, the respondents interviewed at that facility were assigned a higher (lower, respectively) weight to compensate for the underrepresentation (overrepresentation, respectively). More details on the data collection and the design of the survey could be found in Supplementary Figures S1–S3 and in [23].

To measure PTSD we followed [9, 24] and constructed a score based on a two-step procedure. First, respondents were asked whether they had ever experienced a traumatic event. This variable is referred as follow: “sometimes things happen to people that are unusually or especially frightening, horrible, or traumatic. For example,: a serious accident or fire, a physical or sexual assault or abuse, an earthquake or flood, a war, seeing someone be killed or seriously injured, having a loved one die through homicide or suicide”. Those who answered positively were then asked the five following questions: over the past month: (1) Have you had nightmares about the event(s) or thought about them when you did not want to? (2) Have you tried hard not to think about the event(s) or avoided situations that reminded you of them? (3) Have you been constantly on guard, watchful, or easily startled? (4) Have you felt numb or detached from people, activities, or your surroundings? (5) Have you felt guilty or unable to stop blaming yourself or others for the event(s) or for any problems they may have caused? Respondents who answered positively to at least three of these questions were considered as having PTSD. This threshold was chosen because it minimizes false negative screening results, which is especially important in primary care settings to facilitate early detection and treatment of PTSD cases that might otherwise go unrecognized. With a cutoff of 3, [24] identified 94.8% of participants diagnosed with PTSD using the MINI (Mini International Neuropsychiatric Interview). In contrast, a cutoff of 4 identified 82.6%, and a cutoff of 5 only 56.2% of diagnosed cases. Our PTSD variable is a binary variable equal to 1 if individuals have such trouble, and 0 otherwise. This constitutes our main dependent variable. The distributions of responses to these variables are presented in Table 1.

**TABLE 1:** Construction and distribution of the post-traumatic stress disorder variable (France, 2019).

Variables	%	Sample size
Have you experienced a traumatic event?	54,1	1,060
If so, over the past month, have you:		
Had nightmares about the event(s) or thought about the event(s) when you did not want to?	55.3	560
Tried hard not to think about the event(s) or went out of your way to avoid situations that reminded you of the event(s)	59.7	560
Been constantly on guard, watchful, or easily startled?	35.9	560
Felt numb or detached from people, activities, or your surroundings?	19.8	560
Felt guilty or unable to stop blaming yourself or other for the event(s) or any problems the event(s) may have caused?	26.7	560
PTSD: Answered “yes” to at least 3 of the above-mentioned questions	17.2	1,060

Reading: 54.1% of individuals have experienced a traumatic event, and 17.2% developed a post-traumatic stress disorder. All results take survey weights into account.

High-risk health behaviors are measured using self-reported questions on alcohol, tobacco, and cannabis consumption (a binary variable for each variable). These variables will be further used as the other dependent variables.

The other variables used in the study as independent variables are: gender, age, age at migration, region of origin, migration motives, whether entered France without appropriate documentation, the length of stay in France, the level of French proficiency, the type of facility, the type of housing, and the food deprivation. These variables reflect different aspects of the factors that occur before, during, and after migration. Table 2 provides a complete description of all variables and their respective percentage in the analyzed sample (N = 1,060). Table 3 provides a description of both the traumatic event locations and the distribution of PTSD over the other variables.

**TABLE 2:** Descriptive statistics of our sample (France, 2019).

Variables	Description	% in the analyzed sample
Female	Binary variable equal to 1 for women, and 0 for men	29.4
Age at migration	18–25 years old	28.0
	26–30 years old	23.8
	31–31 years old	18.1
	35–46 years old	20.5
	46 years old and more	9.6
Region of origin	Binary variable equal to 1 for those coming from sub-Saharan Africa, and 0 otherwise	62.7
Entered France without appropriate documentation	Binary variable equal to 1 for those entering France without appropriate documentation, 0 otherwise	58.3
Length of stay	Binary variable equal to 1 for those living in France for 5 years or more, and 0 otherwise	28.4
Coming to France for economic reasons	Binary variable equal to 1 for those reported coming to France for economic-related reasons, and 0 otherwise	43.0
Coming to France for health reasons	Binary variable equal to 1 for those reported coming to France for health-related reasons, and 0 otherwise	10.4
Coming to France for political reasons	Binary variable equal to 1 for those reported coming to France for political-related reasons, and 0 otherwise	23.2
Coming to France for family reasons	Binary variable equal to 1 for those reported coming to France for family-related reasons, and 0 otherwise	7.5
Coming to France for security reasons	Binary variable equal to 1 for those reported coming to France for security-related reasons, and 0 otherwise	14.8
Healthcare facilities	Binary variable equal to 1 for facilities providing healthcare services to vulnerable populations, and 0 otherwise	7.9
Level of French proficiency	Binary variable equal to 1 for individuals answering the questionnaire in French and self-reported having a very good level of speaking and reading French	20.4
Food deprivation	Frequent	33,2
	Sometimes	35,4
	Never	31,4
Housing	Living in an apartment	40.3
	Living in a shelter or in social hostel	30.4
	Homeless	29.3
Tobacco	Binary variable equal to 1 if individual reported smoking tobacco every day or occasionally, and 0 if they never smoke tobacco	32.4
Alcohol	Binary variable equal to 1 if individual is an occasional or chronic risk consumer^a^, and 0 otherwise	16.0
Cannabis	Binary variable equal to 1 if individuals reported smoking cannabis at least once per week, and 0 if they never smoke cannabis	14.2

^a^ An occasional risk consumer is defined as a person consuming no more than 21 standardized glasses of alcohol per week for a man (or 14 for a woman), and no more than 6 standardized glasses of alcohol, at most once per month (regardless of gender). A chronic risk consumer is defined as a person consuming at least 22 standardized glasses of alcohol per week for a man (or 15 for a woman) or at least 6 standardized glasses of alcohol per week (regardless of gender). These thresholds are used by the AUDIC-C alcohol consumption questions developed by the WHO [25, 26].

Our analyzed sample contains 1,060 observations. All results take survey weights into account.

**TABLE 3:** Distribution of both the traumatic event locations and the prevalence of post-traumatic stress disorder (France, 2019).

Variables		Traumatic event occurring in			Facing ≥1 traumatic event	PTSD among those facing ≥1 traumatic event	PTSD in the analyzed sample
		Country of origin	Migration journey	France			
Female		26.6	23.1	36.2	28.8	36.8	19.5
At migration	18–25 years	29.5	37.5	31.4	29.3	36.8	20.8
	26–30 years old	24.0	32.3	26.5	26.9	33.5	20.5
	31–35 years old	17.1	17.5	12.3	16.5	22.7	11.1
	36–45 years old	23.7	11.8	23.6	22.3	29.1	17.1
	46 years old and more	5.7	0.9	6.2	4.5	37.3	10.3
Region of origin	Sub-Saharan Africa	68.1	86.1	65.5	71.5	34.4	21.1
Entered France without appropriate documentation		58.9	89.6	65.3	66.1	36.3	22.2
Came in France for	Economic reasons	31.3	36.9	36.2	35.8	18.5	8.0
	Health reasons	33.5	31.4	27.7	30.2	39.7	20.1
	Political reasons	7.2	4.4	9.6	7.7	35.9	24.2
	Family reasons	19.4	29.5	16.5	21.5	27.5	14.3
	Security reasons	10.4	6.9	10.2	9.6	46.2	35.6
Food deprivation	Frequent	22.2	19.1	19.8	22.9	38.0	25.1
	Sometimes	43.0	32.9	41.8	38.7	31.9	18.9
	Never	34.8	47.9	38.4	38.4	21.4	7.9
Housing	Regular apartment	34.9	22.4	35.9	34.0	22.9	10.5
	Shelter or social hostel	35.0	33.4	36.5	33.9	38.8	23.3
	Homeless	30.0	44.2	27.5	32.1	34.1	20.2

Reading: Among undocumented immigrants facing a traumatic event that occurred in the country of origin, during the migration journey, or in France, 26.6%, 23.1% and 36.2% were women. The prevalence of PTSD is 36.8% for women experiencing at least one traumatic event. The prevalence of PTSD is 19.5% for women in our sample. All results take survey weights into account.

Analyses relied on several multivariate probit models. In our main specification, we used PTSD as the dependent variable, and the other variables are used as independent variables. In a second specification, we used high-risk health behaviors as dependent variables (three separate regressions), and PTSD along with the other variables as independent variables. All estimates use survey weights and estimate robust standard errors, accounting for potential heteroskedasticity across surveyed facilities and the types of services provided.

## Results

Results of the multivariate probit models are presented in Tables 2, 3. Tables reported marginal effects, which allows the coefficients to be interpreted in percentage points (pp).

Table 4 estimated how demographics (column 1), migration characteristics (column 2), and living conditions in France (column 3) are correlated with PTSD. These sets of variables are included one after the other in the table. Results showed that among the factors that increase the probability of experiencing PTSD, the strongest is facing food deprivation often and sometimes (+9.2 pp and +7.6 pp, respectively). Migrating for security reasons is also strongly associated with a higher probability of experiencing PTSD (+7.4 pp) as is entering France without appropriate documentation (+6.9 pp). Homeless undocumented immigrants and those living in shelter or in social hostels were also more likely to experience PTSD compared to those living in regular appartements (+5.1 pp and +6.2 pp, respectively). It is worth noting that individuals migrating for economic reasons are less likely to experience PTSD (-11.5 pp).

**TABLE 4:** Factors contributing to the development of post-traumatic stress disorder (France, 2019).

Variables	Probability of experiencing a PTSD			
Marginal effects	(1)	(2)	(3)	(4)
Female	0.0460**	0.0701***	0.0438	0.0413
​	(0.0180)	(0.0265)	(0.0293)	(0.0285)
Ref. age at migration:<26 years Age at migration: 26–30 years	−0.00974	0.00652	0.0197	0.0265
​	(0.0323)	(0.0242)	(0.0200)	(0.0179)
Age at migration: 31–35 years	−0.104***	−0.0833**	−0.0707**	−0.0646**
​	(0.0356)	(0.0418)	(0.0342)	(0.0309)
Age at migration: 36–46 years	−0.0436	−0.0162	−0.0248	−0.0177
​	(0.0523)	(0.0536)	(0.0398)	(0.0467)
Age at migration: >46 years	−0.115***	−0.0756***	−0.0524***	−0.0321***
​	(0.0227)	(0.00885)	(0.0159)	(0.0124)
Region of origin: Sub-saharan Africa	​	0.0618**	0.00812	0.000350
​	​	(0.0264)	(0.0140)	(0.0182)
Length of stay: 5 years and more	​	0.00309	−0.00354	0.0107
​	​	(0.0468)	(0.0377)	(0.0220)
Entered France without appropriate documentation	​	0.102***	0.0943***	0.0693***
​	​	(0.0150)	(0.0191)	(0.0220)
Very good level of French proficiency	​	−0.0182	−0.0153	−0.0164
​	​	(0.0248)	(0.0210)	(0.0193)
Came for economic reasons	​	​	−0.121***	−0.115***
​	​	​	(0.0114)	(0.00869)
Came for political reasons	​	​	0.0608	0.0494
​	​	​	(0.0429)	(0.0356)
Came for family reasons	​	​	−0.0204	−0.00321
​	​	​	(0.0426)	(0.0386)
Came for security reasons	​	​	0.103*	0.0741*
​	​	​	(0.0602)	(0.0439)
Came for health reasons	​	​	0.0571	0.0496
​	​	​	(0.0691)	(0.0541)
Healthcare facilities	​	​	​	0.0251**
​	​	​	​	(0.0103)
Ref. Food deprivation: never Food deprivation: sometimes	​	​	​	0.0766***
​	​	​	​	(0.0153)
Food deprivation: often	​	​	​	0.0926*
​	​	​	​	(0.0481)
Ref. Housing: regular appartment Housing: shelter or social hostel	​	​	​	0.0621***
​	​	​	​	(0.0134)
Housing: homeless	​	​	​	0.0517***
​	​	​	​	(0.0133)
Observations R-squared	1,060 0.017	1,060 0.052	1,060 0.109	1,060 0.131

Reading: Undocumented immigrants who often face food deprivation have an increased probability of 9.2 percentage points to experience a PTSD, by comparison to those never facing such a deprivation.

Coefficients are marginal effects computed after probit models. All results take survey weights into account.

*indicates p < 0.1, **indicates p < 0.05, ***indicates p < 0.01.

It is worth noting that recently arrived individuals might exhibit more pronounced symptoms due to fresh arrival and higher stress levels for being in a new environment. We, therefore, provide a robustness check using the length of stay measured by a categorical variable distinguishing between in France for less than 2 years, from 3 to 5 years, and for more than 5 years (Supplementary Table A2). We also have provided additional results investigating the correlations between PTSD and other mental health symptoms (Supplementary Table A3).

Table 5 followed the same pattern as Table 4 with a focus on the associations between PTSD and three high-risk health behaviors: alcohol, tobacco and cannabis consumptions. The analysis relied on the same sets of demographics, migration characteristics, and living conditions as displayed in column 4 of Table 4. Results showed that PTSD is positively and significantly associated the probability to be an occasional or a chronic risk alcohol consumer (+5.1 pp), but not with tobacco and cannabis consumption.

**TABLE 5:** Association between post-traumatic stress disorder and high-risk health behaviors (France, 2019).

Variables	Alcohol	Tobacco	Cannabis
Marginal effects	(1)	(2)	(3)
PTSD	0.0516***	0.00340	−0.0127
​	(0.0149)	(0.0259)	(0.0152)
Female	−0.126***	−0.152***	−0.118***
​	(0.00708)	(0.0221)	(0.00568)
Ref. age at migration: <26 years Age at migration: 26–30 years	−0.0165	−0.0693**	−0.0859***
​	(0.0121)	(0.0333)	(0.0260)
Age at migration: 31–35 years	0.0138	−0.0972***	−0.0808***
​	(0.0190)	(0.0347)	(0.0197)
Age at migration: 36–46 years	0.0977***	−0.0366	−0.119***
​	(0.0227)	(0.0249)	(0.0302)
Age at migration: >46 years	−0.0565***	−0.109***	−0.215***
​	(0.0167)	(0.0113)	(0.0311)
Region of origin: Sub-Saharan Africa	−0.195**	−0.263***	−0.199**
​	(0.0832)	(0.0876)	(0.0827)
Length of stay: 5 years and more	0.0380	0.0912**	0.0968***
​	(0.0244)	(0.0378)	(0.0248)
Entered France without appropriate documentation	0.0272	0.0513	0.0434
​	(0.0313)	(0.0471)	(0.0287)
Very good level of French proficiency	−0.0142	−0.0799***	−0.0437
​	(0.0164)	(0.0242)	(0.0577)
Came for economic reasons	−0.0330*	−0.00236	0.0347*
​	(0.0186)	(0.0148)	(0.0209)
Came for political reason	0.0555*	−0.0288	0.0190
​	(0.0302)	(0.0278)	(0.0248)
Came for familly reasons	0.167**	−0.0424	0.0664
​	(0.0656)	(0.0718)	(0.0939)
Came for security reasons	−0.000823	−0.0218	0.0352
​	(0.0156)	(0.0207)	(0.0273)
Came for health reasons	−0.0161	−0.142***	0.00566
​	(0.0284)	(0.0460)	(0.0276)
Healthcare facilities	0.0245	0.0389***	−0.0400**
​	(0.0235)	(0.00730)	(0.0160)
Ref. Food deprivation: never Food deprivation: sometimes	0.0345	−0.0260	0.00217
​	(0.0356)	(0.0161)	(0.0128)
Food deprivation: often	0.00507	0.0686***	0.0554***
​	(0.0296)	(0.0183)	(0.0162)
Ref. Housing: regular appartment Housing: shelter or social hostel	−0.100***	0.0126	0.0139
​	(0.0197)	(0.0185)	(0.0185)
Housing: homeless	−0.0578	0.102***	0.0620***
​	(0.0431)	(0.0237)	(0.0141)
Observations R-sqaured	1,060 0.161	1,060 0.233	1,060 0.272

Reading: Undocumented immigrants experiencing a PTSD had an increase probability of 5.1 percentage points to be an occasional or chronic risk alcohol consumer.

Coefficients are marginal effects computed after probit models. All results take survey weights into account.

*indicates p < 0.1, **indicates p < 0.05, ***indicates p < 0.01.

## Discussion

Our findings are threefold. First, the prevalence of PTSD among undocumented immigrants living in France is high, reaching 17.2% — at least eight times greater than what is observed in the general French population [27, 28], and much higher than rates reported in other European countries [20, 29]. Second, both pre-migration, and post-migration factors significantly contribute to the development of PTSD. Third, we showed that PTSD is strongly correlated with a high-risk alcohol consumption after arrival in France.

More precisely, we found that, everything else being equal, individuals who migrated for security reasons were more likely than others to develop PTSD. This is similar to the findings showing that negative push-factors such as war, torture, sexual or gender-based violence, or political persecution explained the immigration decision [30–32]. Push-factors are important to consider because individuals are still choose to migrate despite being aware of the risk of fatality occurring during the migration journey and on the uncertainty of finding employment in destination countries [33]. Once in France, social determinants such as housing instability, food insecurity, and alcohol consumption were other important factors significantly associated with the probability of developing PTSD. These results add up to the reasons explaining the unhealthy acculturation effects reported in several other studies on regular immigration [34–37].

Our study faces the following limitations. First, the cross-sectional nature of the data prevents any causal interpretation of our results. Second, important omitted variables might bias our estimates, such as attitudes toward risk. Risk-taking individuals are found to be more likely to migrate [38, 39], are less prone to healthy behaviors [40–42]. Its omission might lead to spurious correlation between PTSD and the other variables. Third, the associations between PTSD and living conditions might suffer from reverse causality issue. Poor living conditions may be a trigger for and a consequence of PTSD. That is, individuals with poor living conditions are more likely to develop PTSD, and those facing PTSD may me more likely to have poor living conditions. Fourth, while our sample is representative of undocumented immigrants attending facilities providing assistance to vulnerable populations in Paris and Bordeaux, it may not be representative of all undocumented immigrants living in France. In particular, our sample mainly consists of young men from sub-Saharan Africa, many of whom have lived in France for only a few years. This limits the generalizability of our results, particularly if there is self-selection by individuals of specific origins into assistance-providing facilities. Fifth, our PTSD measurement is based on self-reported questions, which might not correspond directly to a clinical diagnosis by a healthcare professional. Additionally, some PTSD items—such as ‘being constantly on guard, watchful, or easily startled’—may partly reflect housing instability, which could contribute to the observed association.

Despite these limitations, we believe that our study can bring valuable insights for policy. Public interventions might, for instance, consider prioritizing undocumented immigrants originating from countries with high levels of violence, as they may face elevated risks of PTSD. Administrative services may, likewise, require special attention, as undocumented immigrants applying for asylum are often obliged to tell their stories, a process that can reactivate traumatic memories. Additionally, previous studies relying on the same representative survey have documented limited access to both healthcare services and health insurance among undocumented immigrants, [43, 44]. Because of this underuse of healthcare services, policy interventions could be more effective in addressing other health determinants, such as access to the formal labor market or to improve living conditions. This could result, for example, in the opening of certain labor market segments to undocumented immigrants with temporary working visas in France. This is likely to be a promise step, as were initiatives like the Deferred Action for Childhood Arrivals (DACA) in the U.S., which have improved mental health and health insurance coverage of undocumented immigrants [45]. Finally, improving access to health and administrative services through dedicated assistance centers would also be another possible public intervention. Future research is nevertheless needed to address the full spectrum of factors shaping undocumented immigrants’ mental health and wellbeing.

## Data Availability

The data supporting the findings of this study are not publicly available due to confidentiality and legal restrictions. Access to the data is restricted, and they cannot be shared publicly.
