# Supplementary material for: Post-Traumatic Stress Disorder Among Undocumented Immigrants. Evidence for the Premier-Pas Survey
Source: Int J Public Health. 2026 Apr 15;71:1608844. doi: 10.3389/ijph.2026.1608844 (PMC13124639; doi:10.3389/ijph.2026.1608844)
Supplement: Supplementary file 1 [file Table1.docx]

Table A1: Sampling characteristics of studies focusing on undocumented immigrants and mental health

| **Name** | **Country of destination** | **Sample size** | **Sampling characteristics** | **Country of origin** | **Methods** |
| --- | --- | --- | --- | --- | --- |
| Heeren and al., 2014 | Switzerland | 21 | Convenient (respondents were recruited in churches) | Multi-countries | Descriptive statistics |
| Naimo and al., 2006 | Italy | 82 | Convenient (Respondents were recruited using companies, professional schools, and host centers) | Albania | Descriptive statistics |
| Andersson and al., | Sweeden | 104 | Convenient (respondents are recruited in 3 medical clinics, Doctor of the world, and the Red Cross) | Multi-countries | Linear regression (with 6 explanatory variables) |
| Myhrvold and Smastuen, 2017 | Norway | 90 | Health center for undocumented immigrants | Multi-countries | Descriptive statistics |
| Angeletti and al., 2020 | Italy | 100 | Convenient (respondents were recruited by the Italian Catholic Church) | Eritrea |  |
| Schoevers and al., 2009 | Netherlands | 100 | Purposive: (respondents were recruited in GPs offices, midwives, and churches) | Multi-countries, but only females | Descriptive statistics |
| Teunissen and al., 2015 | Netherlands | 325 | Convenient: (respondents are recruited in general practices) | Multi-countries | Descriptive statistics |
| Sousa and al., 2020 | Spain | 438 | Purposive: (participants have to speak Spanish and are recruitment in meeting rooms in urban hostels, and workplaces and homes) | Morocco, Ecuador, Romania and Columbia | Descriptive statistics |
| Martin and Sashidharan, 2023 | Literature review | 21-438 |  | Multi countries | Literature review |
