# Supplementary material for: Post-Traumatic Stress Disorder Among Undocumented Immigrants. Evidence for the Premier-Pas Survey
Source: Int J Public Health. 2026 Apr 15;71:1608844. doi: 10.3389/ijph.2026.1608844 (PMC13124639; doi:10.3389/ijph.2026.1608844)
Supplement: Supplementary file 3 [file Table2.docx]

Table A2: Robustness checks

|  | Main Results | Robustness check 1 | Robustness check 2 |
| --- | --- | --- | --- |
| VARIABLES | (1) | (2) | (3) |
|  |  |  |  |
| Female | 0.0413 | 0.0376 | 0.0392 |
|  | (0.0285) | (0.0274) | (0.0279) |
| *Ref: age at migration<26 years old*  Age at migration: 26-30 years | 0.0265 | 0.0225 | 0.0214 |
|  | (0.0179) | (0.0164) | (0.0134) |
| Age at migration: 31-35 years | -0.0646** | -0.0651** | -0.0697** |
|  | (0.0309) | (0.0328) | (0.0293) |
| Age at migration: 36-45 years | -0.0177 | -0.0167 | -0.0186 |
|  | (0.0467) | (0.0472) | (0.0457) |
| 5.cat_age_mig : 46 and more | -0.0321*** | -0.0286* | -0.0337 |
|  | (0.0124) | (0.0153) | (0.0221) |
| Region of origin:  Sub-Saharan Africa | 0.000350 | 0.00189 | -0.00203 |
|  | (0.0182) | (0.0171) | (0.0143) |
| *Ref. Length of stay: 2 years or less* Length of stay: 3-5 years |  | -0.0304** | -0.0329* |
|  |  | (0.0140) | (0.0171) |
| Length of stay: 5 years and more |  | 0.0179 | 0.0240 |
|  |  | (0.0306) | (0.0281) |
| Entered France illegally | 0.0693*** | 0.0701*** | 0.0709** |
|  | (0.0234) | (0.0224) | (0.0290) |
| Very good level of French | -0.0164 | -0.0168 | -0.0109 |
|  | (0.0193) | (0.0176) | (0.0163) |
| Came for economic reasons | -0.115*** | -0.117*** | -0.111*** |
|  | (0.00869) | (0.00875) | (0.0106) |
| Came for policital reasons | 0.0494 | 0.0523 | 0.0587* |
|  | (0.0356) | (0.0337) | (0.0327) |
| Came for familly reasons | -0.00321 | 0.000291 | 0.000216 |
|  | (0.0386) | (0.0377) | (0.0318) |
| Came for security reasons | 0.0741* | 0.0782* | 0.0825** |
|  | (0.0439) | (0.0405) | (0.0330) |
| Came for health reasons | 0.0496 | 0.0496 | 0.0589 |
|  | (0.0541) | (0.0526) | (0.0507) |
| Healthcare facilities | 0.0251** | 0.0236** | 0.0339** |
|  | (0.0103) | (0.00988) | (0.0135) |
| *Ref. food deprivation: Never*  Food deprivation: sometimes | 0.0766*** | 0.0758*** | 0.0797*** |
|  | (0.0153) | (0.0156) | (0.0199) |
| Food deprivation: Frequent | 0.0926* | 0.0932** | 0.0994*** |
|  | (0.0481) | (0.0470) | (0.0381) |
| *Ref. Housing: regular appartment*  Housing: shelter or social hostel | 0.0621*** | 0.0595*** | 0.0596*** |
|  | (0.0134) | (0.0129) | (0.0227) |
| Housing : homeless | 0.0517*** | 0.0451*** | 0.0462*** |
|  | (0.0133) | (0.0148) | (0.00953) |
| *Ref. Income: First quintile*  Income: second quintile |  |  | -0.0926*** |
|  |  |  | (0.0244) |
| Income: third quintile |  |  | -0.0735** |
|  |  |  | (0.0349) |
| Income: fourth quintile |  |  | 0.0149 |
|  |  |  | (0.0245) |
| Income: fifth quintile |  |  | 0.0227 |
|  |  |  | (0.0650) |
| Length of stay: 5 years and more | 0.0107 |  |  |
|  | (0.0220) |  |  |
|  |  |  |  |
| Observations | 1,090 | 1,090 | 1,090 |

Note: Column 1 reports our main results, corresponding to column 4 in Table 4. Column 2 reports the first robustness check, substituting the length of stay measured with a binary variable, with a categorical variable. Column 3 reports the second robustness check, adding the quintile of income. All our results are robust to theses specification checks.
