# Supplementary material for: Post-Traumatic Stress Disorder Among Undocumented Immigrants. Evidence for the Premier-Pas Survey
Source: Int J Public Health. 2026 Apr 15;71:1608844. doi: 10.3389/ijph.2026.1608844 (PMC13124639; doi:10.3389/ijph.2026.1608844)
Supplement: Supplementary file 4 [file Table3.docx]

Table A3: Association between PTSD and mental health issues (marginal effects after probit models)

|  | (1) | (2) | (3) | (4) |
| --- | --- | --- | --- | --- |
| VARIABLES | Depression | Poor self-assessed health | Anxiety | Sleeping problems |
|  |  |  |  |  |
| PTSD | 0.130*** | 0.065 | 0.039 | 0.101*** |
|  | (0.023) | (0.060) | (0.050) | (0.032) |
| Length of stay: 5 years and more | 0.142*** | 0.067** | 0.041*** | 0.067*** |
|  | (0.046) | (0.029) | (0.012) | (0.005) |
| Female | -0.025 | -0.038 | 0.031*** | -0.084* |
|  | (0.045) | (0.032) | (0.012) | (0.050) |
| *Age at migration: <26 years old*  Age at migration: 26–30 years old | *Ref.*  -0.063** | *Ref.*  -0.110*** | *Ref.*  -0.016 | *Ref.*  -0.074** |
|  | (0.025) | (0.033) | (0.031) | (0.034) |
| Age at migration: 31–35 years old | 0.033 | -0.203*** | -0.022 | -0.105*** |
|  | (0.076) | (0.035) | (0.052) | (0.028) |
| Age at migration: 35–46 years old | 0.027 | -0.016 | 0.017 | -0.104*** |
|  | (0.021) | (0.072) | (0.033) | (0.027) |
| Age at migration: >46 years old | 0.065 | 0.120*** | -0.038 | -0.093 |
|  | (0.052) | (0.039) | (0.027) | (0.060) |
| Region of origin: Sub-Saharan Africa | 0.020 | 0.127*** | -0.105* | 0.077 |
|  | (0.043) | (0.024) | (0.060) | (0.052) |
| Entered France illegally | 0.003 | 0.004 | 0.058*** | 0.003 |
|  | (0.010) | (0.072) | (0.019) | (0.061) |
| Came for economic reasons | -0.100*** | -0.016 | -0.010 | -0.058** |
|  | (0.023) | (0.043) | (0.016) | (0.030) |
| Came for health reasons | 0.064 | 0.149*** | 0.022 | 0.103*** |
|  | (0.059) | (0.021) | (0.029) | (0.029) |
| Came for political reasons | -0.072** | -0.027 | 0.010 | -0.017 |
|  | (0.030) | (0.046) | (0.019) | (0.015) |
| Came for family reasons | -0.011 | 0.111** | 0.082** | 0.104*** |
|  | (0.013) | (0.050) | (0.038) | (0.027) |
| Came for security reasons | 0.010 | 0.063 | 0.006 | 0.189*** |
|  | (0.034) | (0.060) | (0.029) | (0.070) |
| *Food deprivation: Never*  Food deprivation: Frequent | *Ref.*  0.117*** | *Ref.*  0.197*** | *Ref.*  0.060*** | *Ref.*  0.011 |
|  | (0.021) | (0.060) | (0.012) | (0.027) |
| Food deprivation: Sometimes | 0.066** | 0.059*** | -0.017** | 0.030 |
|  | (0.033) | (0.012) | (0.008) | (0.036) |
| *Housing: Regular Appartement*  Housing: Hostel | *Ref.*  -0.011 | *Ref.*  -0.046 | *Ref.*  0.018 | *Ref.*  0.047 |
|  | (0.102) | (0.049) | (0.031) | (0.082) |
| Housing: Shelter | -0.056 | 0.103*** | -0.063 | 0.032 |
|  | (0.072) | (0.037) | (0.042) | (0.030) |
| Housing: homeless | 0.012 | 0.126 | -0.017 | 0.153** |
|  | (0.068) | (0.139) | (0.040) | (0.062) |
|  |  |  |  |  |
| Observations | 1,065 | 1,065 | 1,062 | 1,047 |

1. Standard errors in parentheses
2. *** p<0.01, ** p<0.05, * p<0.1
