# Supplementary material for: Post-Traumatic Stress Disorder Among Undocumented Immigrants. Evidence for the Premier-Pas Survey
Source: Int J Public Health. 2026 Apr 15;71:1608844. doi: 10.3389/ijph.2026.1608844 (PMC13124639; doi:10.3389/ijph.2026.1608844)
Supplement: Supplementary file 5 [file DataSheet2.docx]

Figure S2: Types of facilities surveyed (France, 2019)


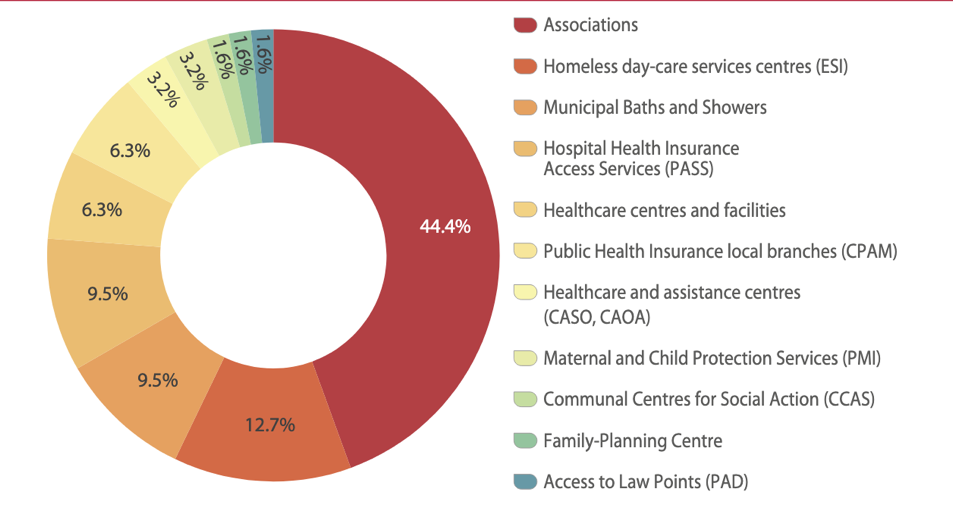


Reading: associations accounted for 44.4% of the facilities providing assistance to vulnerable populations.
