# Supplementary material for: Post-Traumatic Stress Disorder Among Undocumented Immigrants. Evidence for the Premier-Pas Survey
Source: Int J Public Health. 2026 Apr 15;71:1608844. doi: 10.3389/ijph.2026.1608844 (PMC13124639; doi:10.3389/ijph.2026.1608844)
Supplement: Supplementary file 6 [file DataSheet1.docx]

Figure S1: The facilities surveyed (France, 2019)


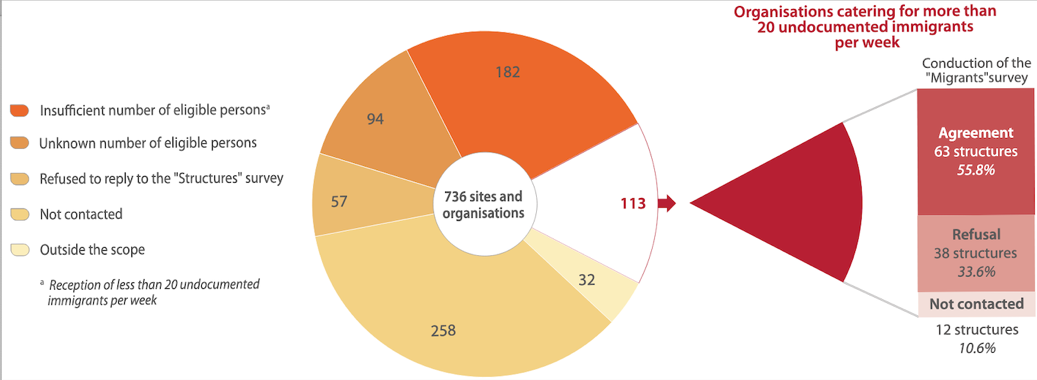


Reading: over the 113 facilities providing assistance to vulnerable populations, 63 (i.e., 56% of them), agreed to participate in the survey.
